# CircRNA circRtn4 as a candidate molecular marker for postmortem interval estimation in mouse brain tissues

**DOI:** 10.3389/fvets.2026.1941625

**Published:** 2026-10-14

**Authors:** Binghui Song, Jie Qian, Jiewen Fu, Zhuohang Xiang, Lin Lu, Tiejun Zhou, Lisha Yang, Jingliang Cheng, Junjiang Fu

**Affiliations:** 1 Key Laboratory of Epigenetics and Oncology, The Research Center for Preclinical Medicine, Southwest Medical University, Luzhou, Sichuan, China; 2 Laboratory of Precision Medicine and DNA Forensic Medicine, the Research Center for Preclinical Medicine, Southwest Medical University, Luzhou, Sichuan, China; 3 Department of Medical Technology, Faculty of Associated Medical Sciences, Chiang Mai University, Chiang Mai, Thailand; 4 Department of Pathology, the Affiliated Hospital of Southwest Medical University, Luzhou, Sichuan, China; 5 Department of Obstetrics and Center for Prenatal Diagnosis, The Affiliated Hospital of Southwest Medical University, Luzhou, China; 6 Laboratory of Forensic DNA, the Judicial Authentication Center, Southwest Medical University, Luzhou, Sichuan, China

**Keywords:** circular RNA (circRNA), forensic biomarkers, postmortem interval (PMI), postmortem RNA degradation, veterinary forensic science

## Abstract

**Introduction:**

Estimation of the postmortem interval (PMI) remains a major challenge in forensic research. Traditional PMI estimation methods using postmortem changes were usually inaccurate or empirical. Owing to their stability, abundance, and tissue specificity, circular RNAs (circRNAs) are promising candidates for PMI estimation. This study explores circRNA markers and aims to develop PMI estimation models in mouse brain tissue, with exploratory modeling in human samples.

**Methods:**

Candidate circRNAs and reference genes were selected from online databases, and primers were designed accordingly. The back-splice junctions of candidate circRNAs were detected by Sanger sequencing. Total RNA was extracted from mouse and human samples collected across different PMIs and temperatures. Semi-quantitative RT-PCR and RT-qPCR were used to quantify circRNAs’ levels, followed by linear and nonlinear regression modeling. Predictive accuracy was assessed using validation samples.

**Results:**

At 25 °C, mRNA degraded faster than the other tested RNAs. According to postmortem stability, 28S rRNA, mt-co1, and circCDR1as were selected as reference genes for the PMI estimation model at room temperature, and 28S rRNA and mt-co1 were selected as reference genes at high temperature. CircRtn4 exhibited PMI-associated changes under both experimental conditions. In semi-quantitative RT-PCR experiments, model comparison supported quadratic models for the 25 °C data normalized to 28S rRNA and circCDR1as, whereas linear models were favored for mt-co1 at 25 °C and for both internal reference at 35 °C. Additionally, cubic terms provided no additional benefit. Validation using known-PMI samples showed variable prediction errors across models and time points. In the RT-qPCR analysis at 25 °C, only the linear model showed a statistically significant relationship with PMI. Exploratory regression equations were fitted to the single-donor human data at 20 °C and 25 °C.

**Discussion:**

CircRtn4 may serve as a molecular marker for PMI estimation in mouse brain tissue under the tested temperature conditions. These findings indicate that postmortem circRNA-based models should be developed under defined temperature and species conditions. Further independent validation in veterinary-relevant species and human cohorts is required before considering forensic application.

## 1 Introduction

In forensic science, the postmortem interval (PMI) generally refers to the interval between death and discovery, examination, or recovery of the body, which can be categorized into early PMI and late PMI. PMI estimation can provide clues for case investigation, delineation of investigation scope, determination of crime time, and screening of alibis. Estimation of PMI has been a hotspot and difficult problem in forensic research, and the accurate estimation of PMI remains a major challenge. Traditional methods for PMI estimation in autopsy cases typically involve estimation of early PMI, such as algor mortis, livor mortis, rigor mortis, autolysis, and autodigestion, as well as estimation of late PMI, such as the degree of putrefaction, forensic entomology, forensic anthropology, etc. (1). These methods were often influenced by environmental and individual variations and were prone to producing false positive or inaccurate results. Traditional methods for PMI estimation to deduce the time of death are relatively short, most within 24 h. In recent years, studies on postmortem degradation patterns of proteins (2), DNA (3), and RNA (4) have provided new methods for inferring PMI.

In the past few years, a variety of RNAs have been explored for PMI estimation, such as ribosomal RNA (rRNA), messenger RNA (mRNA), and microRNA (miRNA), and the results have shown their potential application value for PMI estimation in forensic science (5, 6). With the application and development of techniques such as RNA extraction, reverse transcription, and real-time quantitative polymerase chain reaction (RT-qPCR), many scientists have utilized the characteristics of RNA degradation to estimate PMI (7). Until 2014, there were few studies analyzing the correlation between RNA degradation and PMI, mainly because the standardization of quantitative analysis was difficult to achieve sufficient accuracy and reproducibility. At present, researchers have studied the degradation characteristic of RNA with time of death by using tissues in the heart, brain, kidney, spleen, and lung of mice to provide new experimental evidence for PMI estimation (8–10). However, there are too many uncertain factors affecting the estimation of the time of death, especially susceptible to changes in ambient temperature and environments. Luzhou is located in the southeast of China’s Sichuan Province, where high temperatures and humidity bring difficulties to the PMI estimation. For this reason, only searching for biomarkers that are not easily affected by changes in ambient temperature can bring strong auxiliary evidence for PMI estimation. Circular RNA (circRNA) is currently regarded as an emerging epigenetic marker in a variety of fields. It acts as a sponge for miRNA, participates in protein translation, regulates transcription, and plays a major role in physiology, cancer, immunology, cardiovascular, and metabolic diseases (11–14).

CircRNA, first discovered in the year of 1976 (15), belongs to a class of non-coding RNA (ncRNA) with a closed circular structure which does not have a 5 ′ -end cap structure and a 3 ′ -end Poly (A) tail. It is mainly distributed in exosomes and cytoplasm and is not easily affected by Ribonuclease R (RNase R) (16–18). CircRNA is highly stable in the organism, with tissue, cell type, and developmental-stage specificity (19, 20). Compared with many linear nucleic acids, circRNA has a closed-loop structure, stable expression, and long half-life (21), which might be more suitable for forensic research. At present, researchers tried to explore the application of circRNA in forensic science, such as the estimation of age, the identification of tissues and body fluids, the estimation of bloodstain formation time, and the cause of death analysis (22–25). The characteristics of high stability, high abundance, and tissue specificity give circRNA potential value in the estimation of PMI (26, 27). Research showed that circRNAs were most highly expressed and most specific in the human brain compared with other tissues (28). In addition, circRNAs were enriched and conserved in human and mouse brain tissues, with numerous transcripts abundant in mouse brain also showing high expression in human brain tissue (29). The brain tissue is in a relatively closed anatomical position and is less affected by external factors. Compared with other tissues, tissues such as skin, that are easily exposed to various environments. Thus, brain tissue may have a closer relationship with PMI by controlling temperature as a major influence. Recently, Song et al. investigated the potential of circRNAs in mouse brain tissue as biomarkers for PMI estimation. However, their study did not address the applicability of these markers in human samples. Accordingly, the present study extends this work by examining postmortem alterations of circRNAs in human brain tissue and by establishing corresponding PMI estimation models (30, 31).

This research aims to build PMI estimation models using circRNA to provide a new tool for PMI estimation, with a view to applying it in the practice of forensic medicine. To complete this, brain tissue was selected. The circRNA identification using samples collected from mice (at 25 °C and 35 °C) and humans (at 20 °C and 25 °C) across a range of PMIs. Human and mouse homologous circRNAs were screened in the database and the expression levels of circRNAs were detected by semi-quantitative reverse transcription-polymerase chain reaction (RT-PCR) and reverse transcription quantitative polymerase chain reaction (RT-qPCR). We thus successfully established PMI estimation models by circRtn4 (mmu_circ_0000250) at different temperatures and validated mouse models using known time-of-death samples to prove the accuracy of these models. The homologous human circRTN4 (has_circ_0054598) was additionalamined in an exploratory analysis.

## 2 Materials and methods

### 2.1 Online databases analysis

The circBase database^1^ and circBank database^2^ were searched to select different circRNAs with high or specific expression in mouse brain tissues (29, 32). Meanwhile, the corresponding circRNAs were found in the Tissue-Specific CircRNA Database (TSCD)^3^ to verify the high or specific expression in the brain tissues (33). The National Center for Biotechnology Information (NCBI) website^4^, as well as the Genotype-Tissue Expression (GTEx) database^5^, were used to check the related gene expression profile. We chose to look at the human gene expression profile and calculated the average expression of each gene in brain tissues based on the gene expression data available on the GTEx website. NCBI listed mouse gene expression profile, and the average expression in the cerebellum, frontal lobe, and cortex in brain tissues was calculated. To determine that the selected circRNAs share the same expression profile as the database, expression profiles at the mRNA levels were required in multiple tissues. The expression of circRNA was detected in 13 tissues including the brain, lung, liver, kidney, heart, spleen, stomach, small intestine, straight intestine, testis, uterus, skeletal muscle, and eye. Furthermore, the Internal Control Genes (ICG) 2.0^6^ was used to screen the potential reference gene (34). The literatures of “Cancer Studies,” “Non-cancer Disease Studies”, and “Biological Model” were selected under the “human” species in the ICG database and the usage frequency of all mRNA internal control genes in these studies were counted. To provide a quantitative basis for internal reference selection, temporal preservation of the 11 candidate references at 25 °C was evaluated using the geometric mean ratio of the day-8 signal relative to day 0 with pointwise 95% Welch confidence intervals. Within-time variability was summarized using the median coefficient of variation (CV) across PMI groups. *p*-values for comparisons with day 0 were adjusted using the Benjamini–Hochberg method.

### 2.2 Primers design

Based on the above analysis, we selected circRNAs or internal controls to design primers. The design of circRNA divergent primers was to select the last 200 bp bases and the first >200 bp bases of the circRNA sequence, and the last >200 bp of the circRNA sequence was shifted to the top 200 bp sequence. The “NCBI Blast^7^” was used to design primers for the transformed sequence and ensured that the designed primers are flanking the splice site and that the amplified product spans the splice site (35). The circPrimer (version 1.2.0.0) software was further used to check whether the primers formed circRNA and whether the amplified product spanned the splice site (36). The mRNA primers as well as the circRNA convergent primers were designed according to the conventional primer method (37). Primer sequences are detailed in Supplementary Table 1.

### 2.3 Mouse and human brain tissue samples collection

BALB/c mice were used in this study. All animal experiments were performed in accordance with the international, national, and institutional guidelines for animal care. This study was reviewed and authorized by the Animal Ethics Committee from Southwest Medical University in China (Approval number: 20230821–011 and swmu20250100). A total of 62 BALB/c male mice (9 weeks and 16 weeks, ranging from 23 g to 26 g) in a specific pathogen-free grade were purchased from Tengxin Biotechnology Co., Ltd. (Chongqing, China). Sixteen-week-old mice were used for the 25 °C experiment, whereas 9-week-old mice were used for the 35 °C experiment. According to the standards, the rearing conditions were 20–26 °C, 40–70% humidity, and light and dark alternated for 12 h each. All animals are healthy, do not carry pathogens, have free access to food and water, and are used for equation construction experiments at 25 °C and 35 °C. All mice were sacrificed according to previous study (31). Following sacrifice, the brain tissues were immediately placed in Eppendorf tubes to minimize internal factors and prevent external contamination. These tubes were then transferred to a temperature- and humidity-controlled incubator to simulate different PMIs. The exact post-mortem environmental conditions were strictly maintained at 25 °C and 35 °C (relative humidity 50 ± 5%). Through our preliminary experiment, we found that circRNA was relatively stable in the brain tissue, which was suitable for the study of PMI. So, we selected brain tissue as our first choice among brain, liver, lung, and kidney tissues. For details, about 30-50 mg of whole brain tissue were taken at 0, 1, 2, 4, and 8 d, respectively, after death at 25 °C and taken at 0, 12, 24, 48, 72, and 96 h, respectively, after death at 35 °C. For each time point, five independent mice were used as biological replicates. Following sample quality assessment, four biological samples per PMI were retained for subsequent analysis, resulting in 20 independent biological observations at 25 °C and 24 at 35 °C. In addition, three mice with known times of death were used for verification at each temperature. Meanwhile, 13 fresh tissues including the brain, liver, lung, kidney, heart, spleen, stomach, small intestine, straight intestine, testis, uterus, skeletal muscle, and eye were taken at room temperature.

This study was conducted in strict accordance with the ethical principles outlined in the Declaration of Helsinki and was reviewed and approved by the Ethics Committee of Southwest Medical University Affiliated Hospital. Written informed consent was provided by the legally authorized representative of the deceased donor before the collection of samples and clinical information. For individuals lacking legal capacity, consent was provided by their legal guardians. All biological samples and clinical data were de-identified and used solely for the purposes of this research, ensuring strict protection of participant privacy. Tissue blocks were excised from the cerebral cortex of a healthy male donor with no history of neurological diseases at a baseline PMI of 3 h after death. To precisely evaluate RNA degradation under controlled forensic-relevant conditions, the tissue blocks were placed in EP tubes to prevent internal factors and external contamination. These containers were then transferred to precision environmental incubators set strictly at 20 °C or 25 °C (relative humidity: 50 ± 5%). Following the collection of brain tissue from the forensic autopsy cases, total RNA was extracted at different PMI under two temperature conditions: at 20 °C (3.5 h, 18.5 h, 41.5 h, 64.5 h, 113.5 h) and at 25 °C (3.5 h, 19.5 h, 42.5 h, 65.5 h, 114.5 h). Meanwhile, 3 fresh tissues including the brain, liver, and lung, were taken at room temperature.

### 2.4 Extraction of total RNA and quality control

To minimize pre-analytical variability and operator bias, all tissue processing and RNA extraction procedures strictly followed a standardized operating procedure. The RNA extraction kit (Cat. #: DP419; Tiangen Biotech Co., Ltd., China) was applied to extract total RNA according to the manufacturer’s instructions. The collected RNA was placed on ice for subsequent experiments. The RNA were measured by NanoDrop™ spectrophotometer (Thermo Fisher Scientific Inc., United States), and 260/280 = 1.8–2.0 indicated that the extracted RNA was pure. Total RNA quality were detected by 1% agarose gel electrophoresis, and the integrity and brightness ratio of various rRNA bands were observed to evaluate the integrity and degradation degree of RNA. Extracted RNA was stored at −80 °C for further or directly reverse transcribed into cDNA and stored at −20 °C.

### 2.5 Reverse transcription

ReverTra Ace® qPCR RT Master Mix (Cat. #: FSQ-201, TOYOBO Biotech Co., Ltd., Japan) was used to generate cDNA. A 10 μL reaction system was set up according to the instructions of the kit, and 1 μg RNA was applied for reverse transcription. The reverse transcription reaction was as follows: incubate at 37 °C for 15 min and at 50 °C for 5 min; heat to 98 °C for 5 min. The cDNA was stored at −20 °C after 10-fold dilution for the PCR reaction.

### 2.6 Semi-quantitative RT-PCR

Polymerase chain reaction system containing 2 × Taq PCR Master Mix (Cat. #: KT211-02, Tiangen Biotech Co., Ltd., China) 5 μL, Primer F (10 μM) 0.5 μL, Primer R (10 μM) 0.5 μL, ddH_2_O 3 μL, and cDNA 1 μL. Experiments were executed using an Applied Biosystems Veriti® 96-Well Thermal Cycler PCR (Thermo Fisher Scientific Inc., United States). The procedure is shown in Supplementary Table 2. 2% agarose gel electrophoresis was performed, and exposure imaging was tested by the Bio Rad Universal Hood II (Bio-Rad, United States). In addition, to maintain consistent exposure conditions of the agarose gel electrophoresis, 3 μL of the standard sample with known DNA concentration was applied in each electrophoresis to reduce the influence of exposure conditions. The standard sample was obtained by PCR amplification of genomic DNA (gDNA) from mouse brain tissue using primers of the *Gapdh*. For details, 600 ng gDNA was used for PCR amplification, and the PCR reaction system and procedure are shown in Supplementary Table 3.

### 2.7 RT-qPCR

QuantiNova™ SYBR Green PCR Kit (Cat. #:208154, QIAGEN, Germany) was used for RT-qPCR. According to the instructions, the reaction mixture including 2 × SYBR Green PCR Master Mix 5 μL, QN ROX Reference Dye 1 μL, RNase-Free H_2_O 2 μL, Primer F 0.5 μL, Primer R 0.5 μL, and cDNA 1 μL. Experiments were performed using an Applied Biosystems StepOneplus™ Real-Time PCR System Thermal Cycling Block (Thermo Fisher Scientific Inc., United States). The procedure is shown in Supplementary Table 2. Each sample was analyzed in triplicate, and the difference of standard deviation was less than 0.5 can be used for data analysis.

### 2.8 Construction of mouse model

ImageJ (Version 2.9.0, National Institutes of Health, United States) software was used for the calculation and standardization of the integrated density (IntDen). For details, we first subtracted the IntDen of all bands from the IntDen of the image background to remove the background effect. After removing the influence of exposure condition, the gray values of the standard samples were used to quantify the gray values of the target gene and the internal reference, and the relative level of the target PCR product was calculated by dividing the average grey value of the target gene at each time period by the average grey value of the internal reference bands. After standardizing the gray value of bands on different gels according to the standard samples, the formula of mathematical model based on semi-quantitative RT-PCR is as follows: *K* = (gray value of circRtn4 0-d)/(gray value of internal reference 0-d); *Y* = (gray value of circRtn4 n-d − circRtn4 background gray value)/(gray value of internal reference n-d − internal reference background gray value)/K.

In RT-qPCR experiments, we calculated the average value of reliable data for circRtn4 and 28S rRNA at each time point and corresponding 2^-ΔΔCt^ values. The formulas are as follows: ΔCt = Ct (biomarker) − Ct (internal reference); ΔΔCt = ΔCt (sample) − ΔCt (calibrator, PMI = 0). We established the equation with PMI as the X-axis and 2^-ΔΔCt^ as the Y-axis. The GraphPad Prism 8 (Version 8.0.2, GraphPad Software, United States) software was used to build the linear regression equation and nonlinear regression equation, and the corresponding data were calculated to obtain the *p*-value and *R*^2^ of the equation. Candidate models were compared using adjusted *R*^2^, Akaike information criterion (AIC), Bayesian information criterion (BIC), the *p*-value of the highest-order polynomial term, and leave-one-animal-out cross-validation (LOOCV) mean absolute error (MAE) and root mean squared error (RMSE). Four biologically independent mice were included at each PMI. Lower AIC, BIC, MAE, and RMSE values indicate better model parsimony or predictive performance, respectively. Regression assumptions and model robustness were further assessed for these models. Nested linear, quadratic, and cubic models were additionally compared using partial F tests. Residuals were examined using residual-versus-fitted plots and normal Q–Q plots, and variance structure was assessed using scale-location plots and the Breusch–Pagan test. Residual normality was additionally evaluated using the Shapiro–Wilk test. Potentially influential observations were assessed using studentized residuals and Cook’s distance, with Cook’s D > 4/*n* (total number of observations) used as a screening threshold for their observations. The error rate = ∣ (estimated PMI- real PMI) ∣/ (real PMI) × 100%, which was used to quantify the relative prediction error in the preliminary validation.

### 2.9 Preliminary exploratory analysis in human tissues

Following the successful establishment of the mouse model, the investigation was extended to a human analysis. The homologous human circRNA circRTN4 (hsa_circ_0054598) was identified through database screening and subsequently validated by BLAST analysis. Divergent primers specific to circRTN4 were designed, and its relative expression levels were quantified and further confirmed by Sanger sequencing.

To enable accurate quantification of postmortem changes in human samples, a standardized reference was generated using a standard sample of known concentration. Briefly, cDNA synthesized from 1 μg of total mouse brain RNA was amplified using 28S rRNA primers (Supplementary Table 1). Detailed PCR conditions are provided in Supplementary Table 3. The amplification products were resolved by 1.5% agarose gel electrophoresis, after which the target band was excised and purified using the TIANgel Purification Kit (Cat. #: DP219; Tiangen Biotech Co., Ltd., China) according to the manufacturer’s instructions. The purified DNA was eluted, quantified using a NanoDrop™ spectrophotometer (Thermo Fisher Scientific, United States), and diluted with nuclease-free water to a final concentration of 55 ng/μl for subsequent use.

Semi-quantitative RT-PCR was used to characterize PMI-associated changes in circRTN4, and exploratory regression equations were fitted to the single-donor dataset.

## 3 Results

### 3.1 Circular RNAs selection in brain tissue

Firstly, to select circRNAs for the study, the circRNAs were screened and validated in the circBase and TSCD database. By viewing the host gene expression profiles of circRNA in humans on the GTEx website, genes with relatively high or specific expression in brain tissue were selected, and the average expression in the brain tissues was calculated based on the gene expression data provided by the GTEx website. As shown in Supplementary Figure S1, the expression of selected genes was as follows: RTN4 (196.4 TPM (Transcript per million)), ATP5C1 (92.6 TPM), BCAS1 (44.6 TPM), and BRSK2 (165.9 TPM) (Supplementary Figures S1A–D). These gene expression profiles in mice were searched in the (NCBI) database, as shown in Supplementary Figure S2, and the average expression in the cerebellum, frontal lobe, and cortex of brain tissues were calculated as follows: Rtn4 (48.23 RPKM (Reads per kilobase per million mapped reads)), Atp5c1 (59.67 RPKM), Bcas1 (28.47 RPKM), and Brsk2 (24.57 RPKM) (Supplementary Figures S2A–D). In this study, mmu_circ_0000250 (circRtn4), mmu_circ_0010288 (circAtp5c1), mmu_circ_0009627 (circBcas1), and mmu_circ_0001648 (circBrsk2) were selected as potential biomarkers for further research. Among these biomarkers, circRtn4, circAtp5c1, and circBcas1 were human-mouse homologous and conserved circRNA, which facilitates experimental validation using human samples at a later stage.

### 3.2 Validation of circular RNAs

Convergent and divergent primers were designed for circRNAs and Actb (Supplementary Table 1). Firstly, analysis of the circPrimer software showed that the structures and primers for circRtn4, circAtp5c1, circBcas1, and circBrsk2 did not span the splice site (Figures 1A–D). In the results of amplification using divergent primers, circRtn4, circAtp5c1, circBcas1, and circBrsk2 could be detected as a single band in cDNA, while there was no band at the corresponding position in gDNA (Figure 1E). In addition, we validated these circRNAs by agarose electrophoresis. The divergent primer forms a circRNA band of the corresponding size in the cDNA, but not in the gDNA; and the convergent primer forms a band in the cDNA that is the correct size but different in size from the band detected by the divergent primer (Figure 1F). These results demonstrated that circRNA was reverse loop-forming. Actb is a typical mRNA that cannot be detected by divergent primer, but can be detected by convergent primer, and has the band amplified by convergent primer in both cDNA and gDNA. In these experimental processes, we found that the inconsistency between the size of the bands in gDNA and cDNA by the convergent primer may be due to the inclusion of introns in the amplified products (Figure 1F).

**Figure 1:**
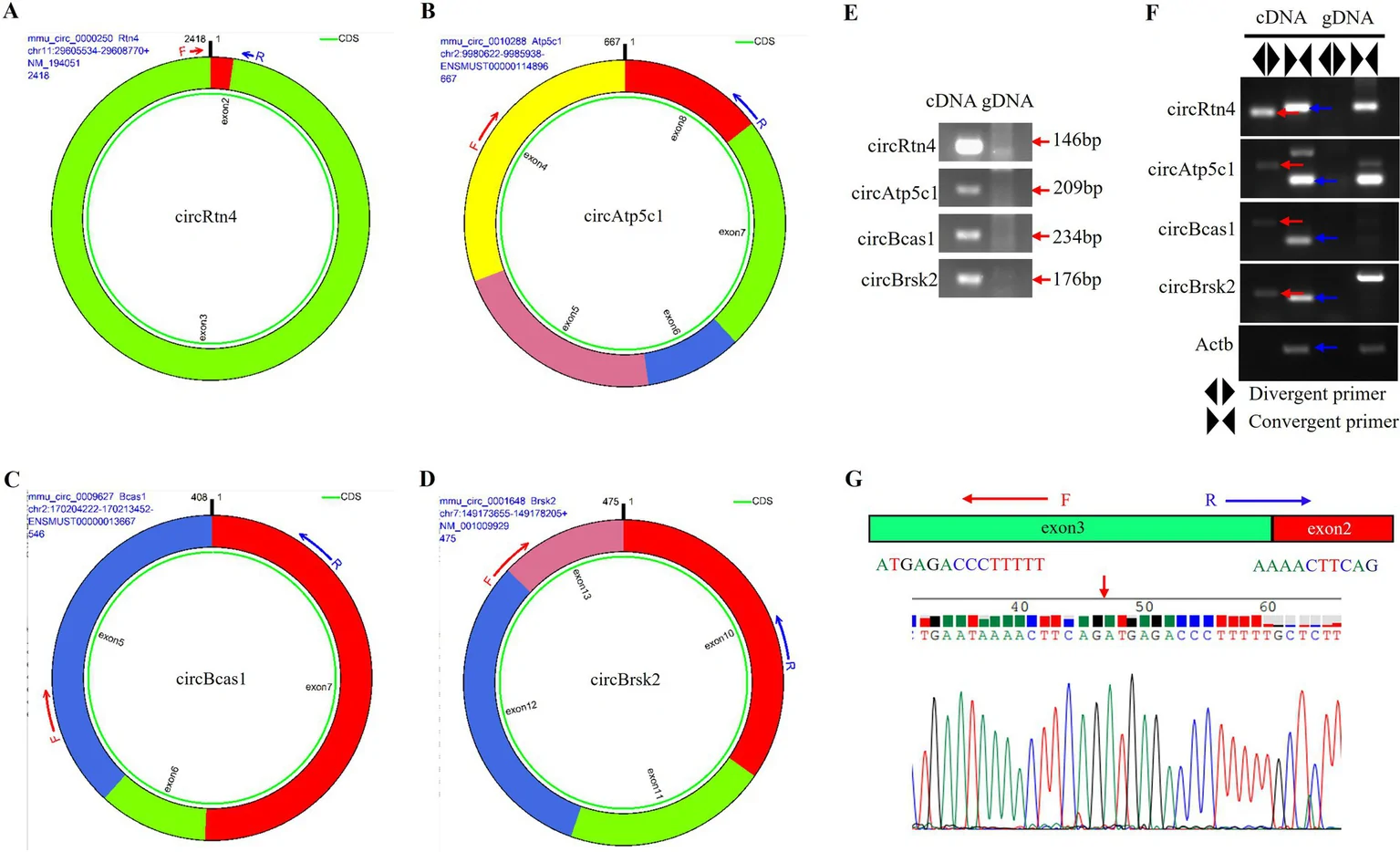
The validation of the selected circRNAs. **(A, B, C, D)** The CircPrimer software showed the primers positions of circRtn4, circAtp5c1, circBcas1, and circBrsk2. **(E)** The electrophoresis results of circRtn4, circAtp5c1, circBcas1, and circBrsk2 in digested cDNA and gDNA. **(F)** The electrophoresis results of convergent and divergent primers of circRtn4, circAtp5c1, circBcas1, and circBrsk2. **(G)** Identification of circRtn4 sequences by Sanger sequencing. F, Forward primer; R, Reverse primer. Red arrows: divergent primer; Blue arrows: convergent primer. Vertical red arrow: the splice sites of exons 2 and 3 of circRtn4.

To confirm whether these circRNAs were suitable for our following PMI estimation model construction, we observed the circRNA levels of 13 tissues including the brain, liver, lung, kidney, heart, spleen, stomach, small intestine, straight intestine, testis, uterus, skeletal muscle, and eye (Supplementary Figure S3). The results demonstrated that circRtn4 was expressed in multiple tissues, and had the highest level in the brain tissue (Supplementary Figure S3). In addition, the level of circAtp5c1 was higher in the brain and heart tissues than in other tissues, while circBcas1 and circBrsk2 specifically existed in the brain tissue (Supplementary Figure S3). The results were mostly consistent with the expression profile of the host gene on the GTEx and NCBI websites. Therefore, circRtn4 with relatively high expression in brain tissue was selected as the potential biomarker in these studies. To verify whether the sequence of circRtn4 was connected head to tail, we performed the Sanger sequencing verification, and the result showed that the amplified product spanned the splice site, demonstrating that the sequences of circRtn4 are correct (Figure 1G).

### 3.3 Reference genes selection results

Reference genes’ selection is critical the stability of the reference gene is very important for the PMI estimation model the reference gene is used to normalize the target gene, but it can also be affected by species, tissue, developmental stage, and other factors. Therefore, it is necessary to select appropriate reference genes through comparison and validation. During the internal references screening process, the ICG website was first used to screen the required internal references, which contains relevant internal references not only for mRNA studies but also for ncRNA studies. The usage frequency of all mRNA reference genes was counted based on the statistical results, we selected *Gapdh*, *Actb*, *Tbp*, *B2m*, *Hprt1*, *Ppia*, and *Rplpo*, which were frequently used, as candidate reference genes (supplementary Figure S4). Furthermore, according to the relevant literatures (9, 38), we selected the commonly used reference genes such as 18S rRNA, 28S rRNA, and circCDR1as (29) for exploration. Mitochondrial mRNA mt-co1 expression is mostly related to their half-lives and are RNases resistant (39). We thus also selected mitochondrial mRNA mt-co1 as a reference gene to PCR amplification. Therefore, a total of eleven candidate internal reference genes were screened for subsequent experiments and primer information is shown in Supplementary Table 1.

The stability of each reference gene was explored by measuring its RNA levels under different PMIs, and the appropriate reference gene was screened. The results of semi-quantitative RT-PCR showed different degradation trends of our selected eleven candidate reference genes (Figure 2A). Based on the relative degradation levels of mouse samples at 0, 1, 2, 4, and 8 d after death at 25 °C, the degradation trend of the eleven candidate references was different with mRNA *Tbp* fast degradation and 28S rRNA, 18S rRNA, circCDR1as and mt-co1 degraded slowly over time compared with other tested mRNAs (Figure 2B). Among 28S rRNA, 18S rRNA, circCDR1as, and mt-co1, the 18S rRNA was degraded too quickly after 4 d. The half-life of 18S rRNA, 28S rRNA, circCDR1as and mt-co1 were 6.9 d, 9.5 d, 8.6 d, and 8.9 d, respectively, indicating the half-life of 18S rRNA is shorter than that of the others. So, circCDR1as, 28S rRNA, and mt-co1 were applied as internal references in semi-quantitative RT-PCR at 25 °C (Figure 2). Quantitative evaluation of the semi-quantitative RT-PCR data further showed that 28S rRNA, circCDR1as, and mt-co1 retained approximately 58–59% of their day-0 signal at day 8, whereas most conventional mRNA candidates showed substantially greater reductions (Supplementary Table 4; Figure S5). Their median within-time CVs were 7.36%, 13.02%, and 6.44%, respectively. These RNAs were therefore retained as comparatively better-preserved reference candidates rather than assumed to be invariant housekeeping controls.

**Figure 2:**
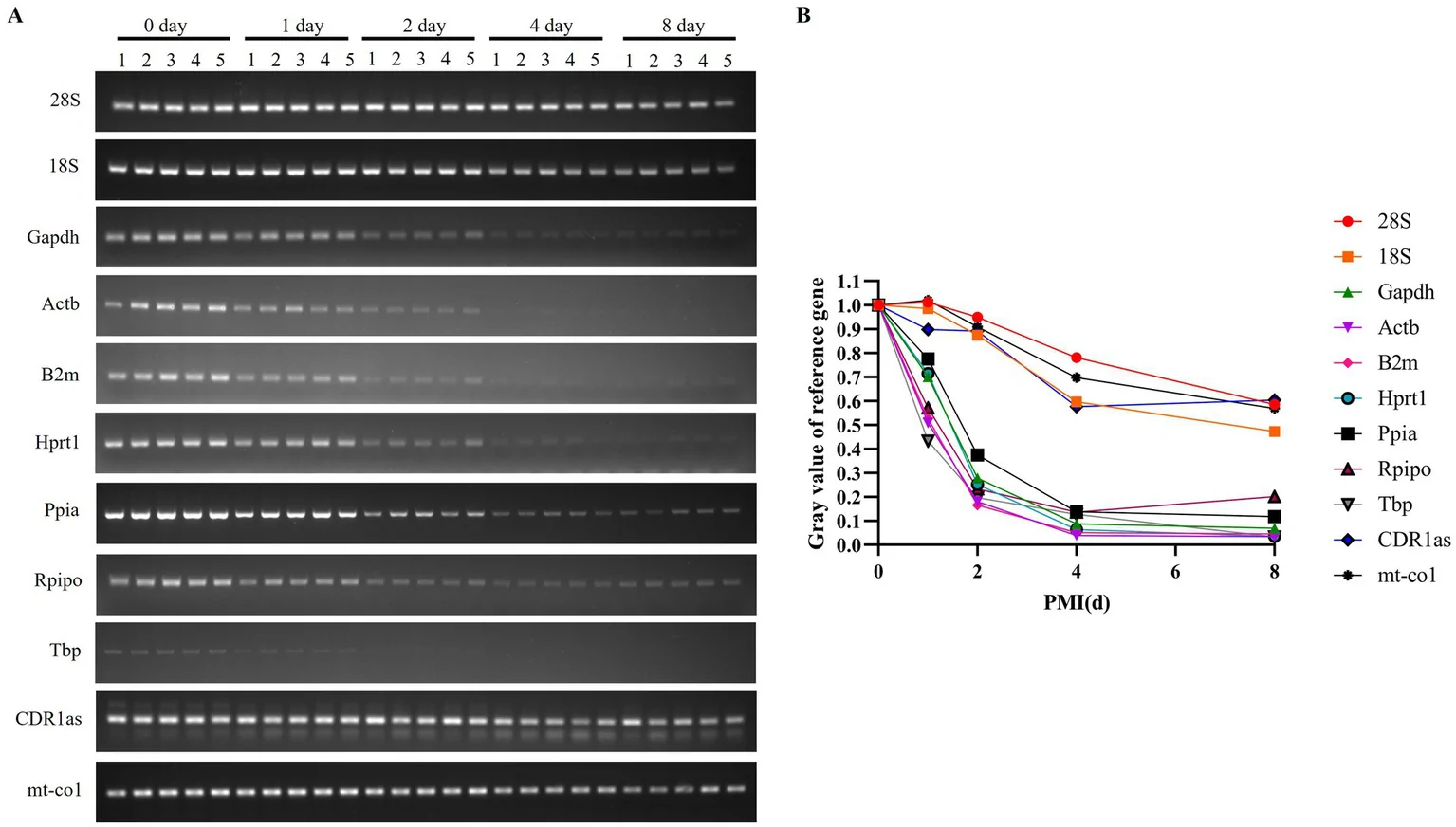
RNA level of reference genes in the brain tissue at 0, 1, 2, 4, and 8 day(s) at 25 °C. **(A)** RNA level of the reference genes at different time periods in semi-quantitative RT-PCR experiment. **(B)** Quantitative results of panel **A** relative to 0 day.

As for under high-temperature conditions of 35 °C, circCDR1as was degraded too quickly (data not shown), so 28S rRNA and mt-co1 were also applied as the internal reference by semi-quantitative RT-PCR at 35 °C. In conclusion, circCDR1as, 28S rRNA, and mt-co1 were used as internal references in semi-quantitative RT-PCR at 25 °C, and 28S rRNA and mt-co1 were selected as the reference genes for semi-quantitative RT-PCR at 35 °C.

### 3.4 Establishment of the mouse PMI estimation models by semi-quantitative RT-PCR

To build a mathematical model at 25 °C, RNA was extracted from the brain tissue samples for 0, 1, 2, 4, and 8 d at 25 °C treatments after death, respectively. The results of 1% agarose gel electrophoresis indicated that the 28S rRNA and 18S rRNA bands were relatively complete or very bright on 0, 1, and 2 d after death, and the RNA degradation was obvious on 4 and 8 d after death (Figure 3A). CircCDR1as, 28S rRNA, and mt-co1 were applied as internal references at 25 °C, semi-quantitative experiments with circRtn4 and internal control were performed (Figure 3B). The equations were developed based on the ratio of biomarker gray values to internal reference gray values at different time periods (Figures. 3C–E). There are two unknown factors, Y: (gray value of circRtn4 n-d − circRtn4 background)/(gray value of internal reference n-d − internal reference background)/K; X: PMI. Taking the ratio of the gray value of the biomarkers to the gray value of the internal reference on 0-d as the K value, and the K values for circRtn4/circCDR1as, circRtn4/28S rRNA and circRtn4/mt-co1 were 0.5716, 0.6718 and 0.9055, respectively.

**Figure 3:**
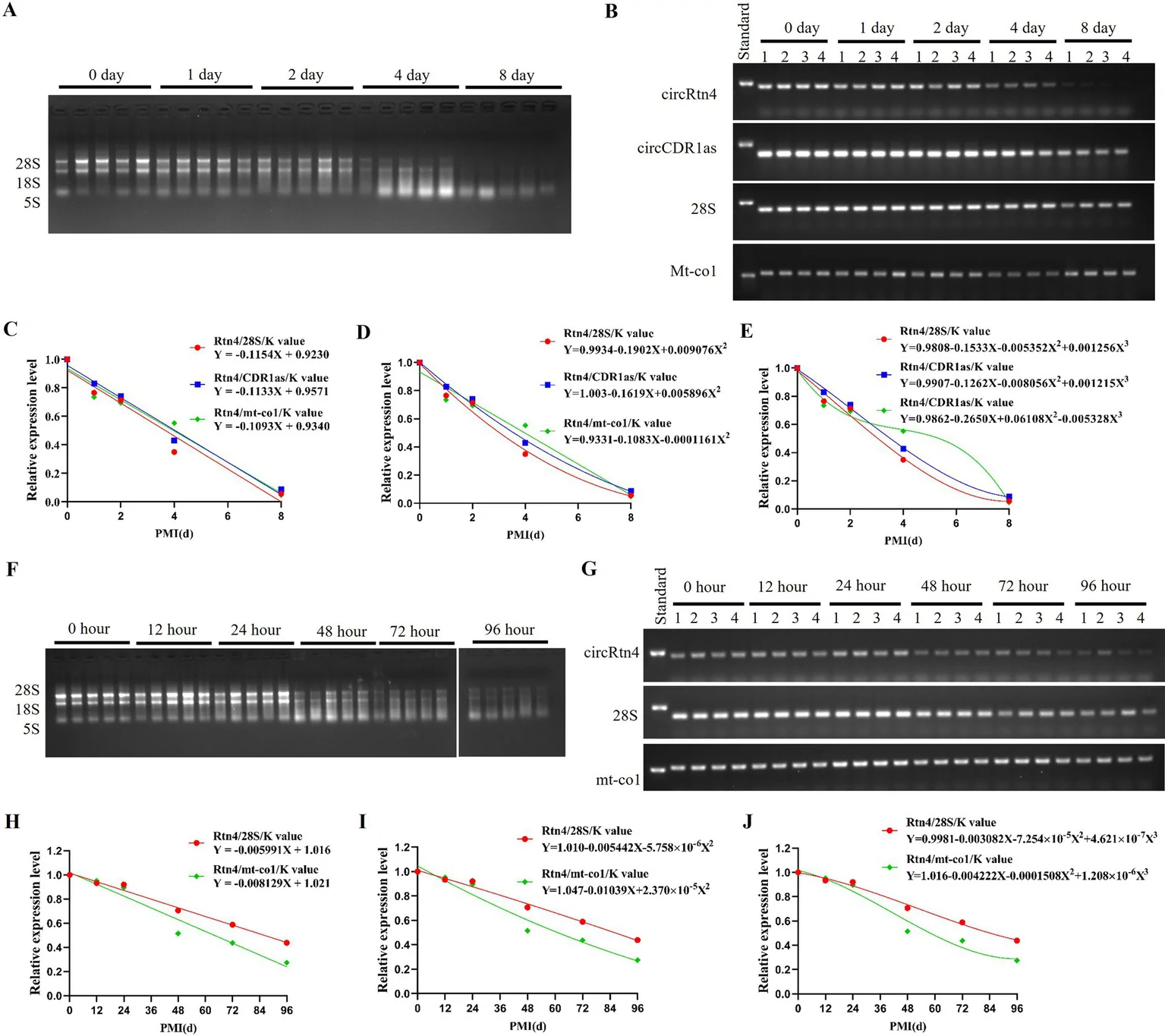
Establishment of mouse PMI estimation model by 25 °C and 35 °C equation, respectively. **(A)** Electrophoretic quality of RNA extraction from the brain tissue of BALB/c mice at 25 °C. **(B)** The plots of semi-quantitative RT-PCR electrophoresis results for circRtn4, circCDR1as, 28S rRNA, and mt-co1 at 25 °C. **(C–E)** The linear, quadratic, and cubic equations were built at 25 °C. In these panels, the colors indicate the specific internal reference used for normalization such as red circles represent 28S rRNA, blue squares represent circCDR1as, and green diamonds represent mt-co1. **(F)** Electrophoretic quality of RNA extraction from the brain tissue of BALB/c mice at 35 °C. **(G)** The plots of semi-quantitative RT-PCR electrophoresis results for circRtn4, 28S rRNA, and mt-co1 at 35 °C. **(H–J)** The linear, quadratic, and cubic equations were built at 35 °C. In these panels, red circles represent 28S rRNA, and green diamonds represent mt-co1. PMI: postmortem interval.

At 35 °C, we extracted RNA from the brain tissue samples at 0, 12, 24, 48, 72, and 96 h after death. The findings of gel electrophoresis indicated that 28S rRNA and 18S rRNA were relatively complete or very bright at 0, 12, 24, 48, and 72 h, and slightly degraded at 96 h. Although the bands of 28S rRNA and 18S rRNA existed at each time point, RNA was more easily degraded at 35 °C (Figure 3F). We thus chose 28S rRNA as an internal reference and circRtn4 as a biomarker at 35 °C (Figure 3G). The results revealed that mt-co1 was more stable than circCDR1as and similar to the stability of 28S rRNA, so we did not choose circCDR1as due to less stability at 35 °C (Data not shown). The ratio of the gray value of the biomarkers to the internal reference at various time periods was calculated and transformed into equations (Figures 3H–J). After calculation, the *K* values for circRtn4/28S rRNA and circRtn4/mt-co1 were 0.4475 and 0.5455, respectively.

When circCDR1as was used as a reference gene at 25 °C, the R^2^ and the *p* values of the three equations were 0.9823 (*p* = 0.001), 0.9957 (*p* = 0.004), and 0.997 (*p* = 0.070), and the *p* values of the linear equation and the quadratic equation were less than 0.05. When 28S rRNA was applied as a reference gene at 25 °C, the R^2^ and the *p* values of the three equations were 0.9571 (*p* = 0.004), 0.9869 (*p* = 0.013), and 0.9882 (*p* = 0.138), and the *p* values of the linear equation and the quadratic equation were less than 0.05. When mt-co1 was used as a reference gene at 25 °C, the R^2^ and the *p* values of the three equations were 0.9673 (*p* = 0.003), 0.9673 (*p* = 0.033), and 0.9931 (*p* = 0.105), and the *p* values of the linear equation and the quadratic equation were less than 0.05. When 28S rRNA was applied as a reference gene at 35 °C, the *R*^2^ and the *p* values of the three equations were 0.9870 (*p* < 0.0001), 0.9880 (*p* = 0.001), and 0.9910 (*p* = 0.014), and the *p* values of the three equations were less than 0.05. The *R*^2^ values of all three equations were close to 0.99 and *p* < 0.05. When mt-co1 was used as a reference gene at 35 °C, the R^2^ and the *p* values of the three equations were 0.9564 (*p* = 0.001), 0.9619 (*p* = 0.007), and 0.9717 (*p* = 0.042), and the *p* values of the three equations were less than 0.05. The *R*^2^ values of all three equations were close to 0.96–0.97 and *p* < 0.05 (Table 1).

**Table 1:** Establishment of mathematical models by semi-quantitative RT-PCR.

Temperature	Internal reference	Regression model	Equation	R^2^	*p*
25 °C	circCDR1as	Linear	Y = −0.1133X + 0.9571	0.9823	0.001
		Quadratic	Y = 1.003–0.1619X + 0.005896×^2^	0.9957	0.004
		Cubic	Y = 0.9907–0.1262X-0.008056×^2^ + 0.001215×^3^	0.9970	0.070
	28S rRNA	Linear	Y = -0.1154X + 0.9230	0.9571	0.004
		Quadratic	Y = 0.9934–0.1902X + 0.009076×^2^	0.9869	0.013
		Cubic	Y = 0.9808–0.1533X-0.005352×^2^ + 0.001256×^3^	0.9882	0.138
	Mt-co1	Linear	Y = -0.1093X + 0.9340	0.9673	0.003
		Quadratic	Y = 0.9331–0.1083X-0.0001161×^2^	0.9673	0.033
		Cubic	Y = 0.9862–0.2650X + 0.06108×^2^-0.005328×^3^	0.9931	0.105
35 °C	28S rRNA	Linear	Y = −0.005991X + 1.016	0.9870	0.000
		Quadratic	Y = 1.010–0.005442X-5.758 × 10^−6^×^2^	0.9880	0.001
		Cubic	Y = 0.9981–0.003082X-7.254 × 10^−5^×^2^ + 4.621 × 10^−7^×^3^	0.9910	0.014
	Mt-co1	Linear	Y = −0.008129X + 1.021	0.9564	0.001
		Quadratic	Y = 1.047–0.01039X + 2.370 × 10^−5^×^2^	0.9619	0.007
		Cubic	Y = 1.016–0.004222X-0.0001508×^2^ + 1.208 × 10^−6^ ×^3^	0.9717	0.042

CircCDR1as degraded too rapidly at 35 °C, hence its absence in the 35 °C group.

Linear, quadratic, and cubic candidate equations were initially fitted for each internal reference and experimental condition (Table 1). Final model selection was based on AIC, BIC, LOOCV, and nested-model F tests rather than *R*^2^ alone. Quadratic models were supported for the 25 °C data normalized to 28S rRNA and circCDR1as, whereas linear models were favored for mt-co1 at 25 °C and for both reference at 35 °C (Supplementary Table 5). Addition of a quadratic term significantly improved the 25 °C 28S rRNA and circCDR1as models (*p* = 0.0077 and 0.0338, respectively), whereas cubic terms provided no further improvement. Regression diagnostics were generally acceptable for the selected models, although some departure from residual normality was observed for the 25 °C mt-co1 model and mild heteroscedasticity was detected for the 35 °C 28S rRNA model (Supplementary Tables 6, 7, and Figure S6).

### 3.5 Establishment of the mouse PMI estimation model by RT-qPCR

We further constructed a PMI estimation model by RT-qPCR at 25 °C. The fluorescence quantitative melting curves identified single peaks of circRtn4, circCDR1as, and 28S rRNA, showing that the primers were specifically amplified (Figures 4A–C). This study presented a linear plot of the amplification curve, where the normalized reporter (Rn) was the ratio of the fluorescence emission intensity to the reference dye. Furthermore, ∆Rn was the normalized result of subtracting the baseline from Rn. The amplification curves showed consistent amplification profiles across retained reactions (Figure 4D).

**Figure 4:**
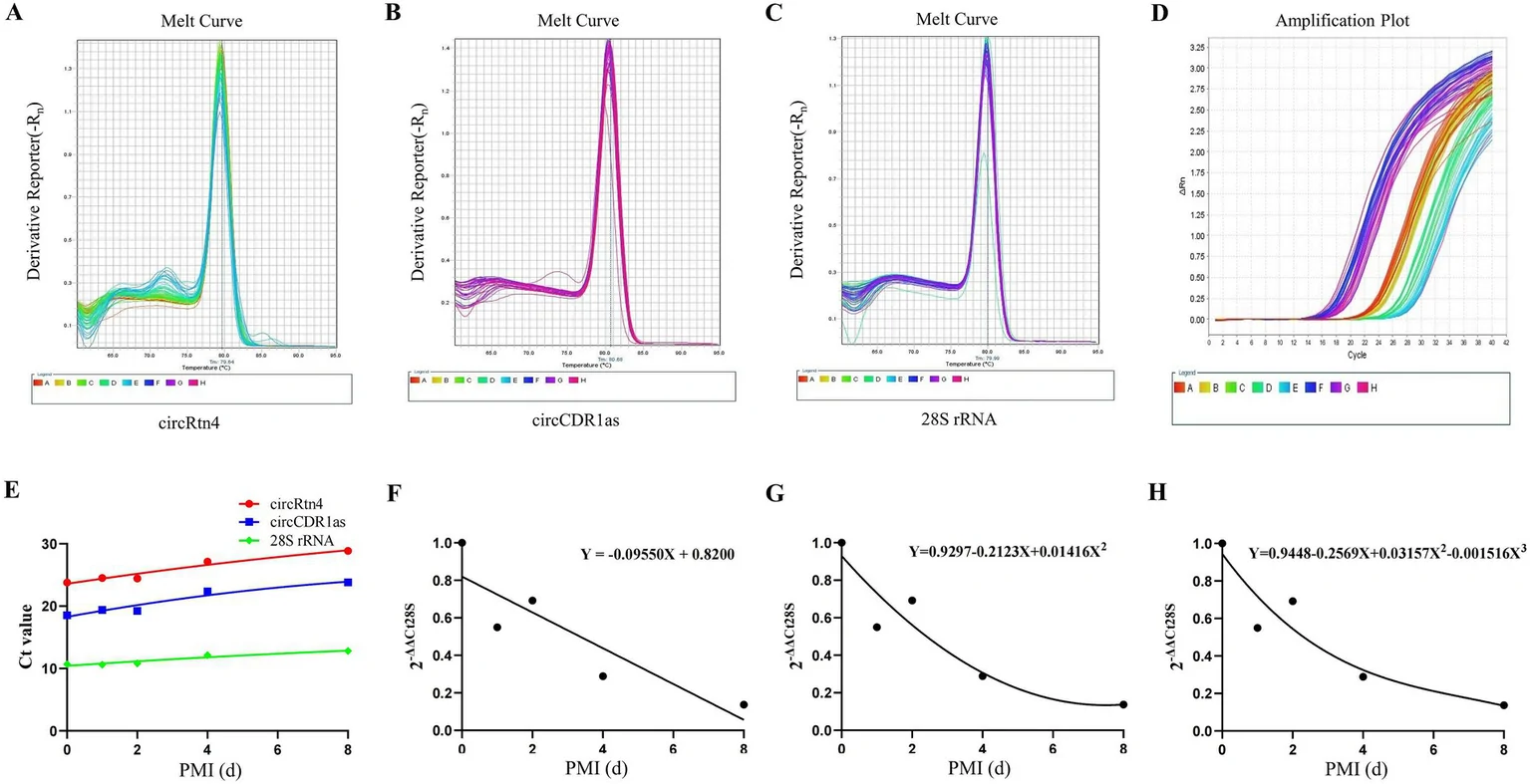
The results of RT-qPCR experiments. **(A–C)** The melting curves of circRtn4, circCDR1as, and 28S rRNA, respectively. **(D)** The amplification curves with the abscissa as the cycle number and the ordinate as the change of fluorescence intensity. **(E)** Relationship between Ct values of circRtn4, circCDR1as, 28S rRNA, and PMI. In this panel, the colors distinguish the target genes such as red circles represent circRtn4, blue squares represent circCDR1as, and green triangles represent 28S rRNA. **(F–H)** CircRtn4 and 28S rRNA for the construction of linear equation, quadratic equation, and cubic equation, respectively. There are two unknowns, including Y: 2^-ΔΔCt^ and X: PMI. Ct: Cycle Threshold.

We visualized the Ct values of circRtn4, circCDR1as, and the internal reference 28S rRNA changed with PMI (Figure 4E). As for RT-qPCR at 25 °C conditions, based on the linear relationship between Ct value and PMI, the result indicated that the slopes of the degradation curves for circRtn4 and circCDR1as were 0.6661 and 0.6956, respectively. The degradation trends of circRtn4 and circCDR1as were found to be almost the same, indicating that circCDR1as was not suitable as an internal reference at 25 °C conditions. Therefore, only 28S rRNA was identified as the reference gene by RT-qPCR at 25 °C to establish mathematical models (Figures 4F–H). The *R*^2^ and the *p*-values of the three equations were 0.792 (*p* = 0.043), 0.8797 (*p* = 0.120), and 0.8819 (*p* = 0.429), respectively. The R^2^ values of the three equations were close to 0.80–0.90, but only the *p* value of the linear equation was less than 0.05 (Table 2). This result indicated that the PMI estimation model based on RT-qPCR could be established by the linear equation.

**Table 2:** Establishment of mathematical models by RT-qPCR at 25 °C.

Internal reference	Regression model	Equation	*R^2^*	*p*
28S rRNA	Linear	Y = -0.09550X + 0.8200	0.7920	0.043
	Quadratic	Y = 0.9297–0.2123X + 0.01416×^2^	0.8797	0.120
	Cubic	Y = 0.9448–0.2569X + 0.03157×^2^-0.001516×^3^	0.8819	0.429

### 3.6 Validation of the PMI estimation model

For the preliminary validation, we used samples with known PMI (2 d and 4 d) to validate the mathematical model of the semi-quantitative PCR experiment at 25 °C (Figures 5A, B). When circCDR1as was used as the internal reference, the error rates of the 2 d validation samples for the three equations were 75%, 55%, and 55%, respectively; the error rates of the 4 d validation samples for the three equations were 17.5%, 7.5%, and 5.0%, respectively. When 28S was used as the internal reference, the error rates of the 2d validation samples for the three equations were 65%, 35%, and 40%, respectively; the error rates of the 4 d validation samples for the three equations were 7.5%, 10%, and 10%, respectively (Table 3). When mt-co1 was used as the internal reference, the error rates of the 2 d validation samples for the three equations were 20%, 20%, and 10%, respectively; the error rates of the 4 d validation samples for the three equations were 10%, 10%, and 48%, respectively.

**Figure 5:**
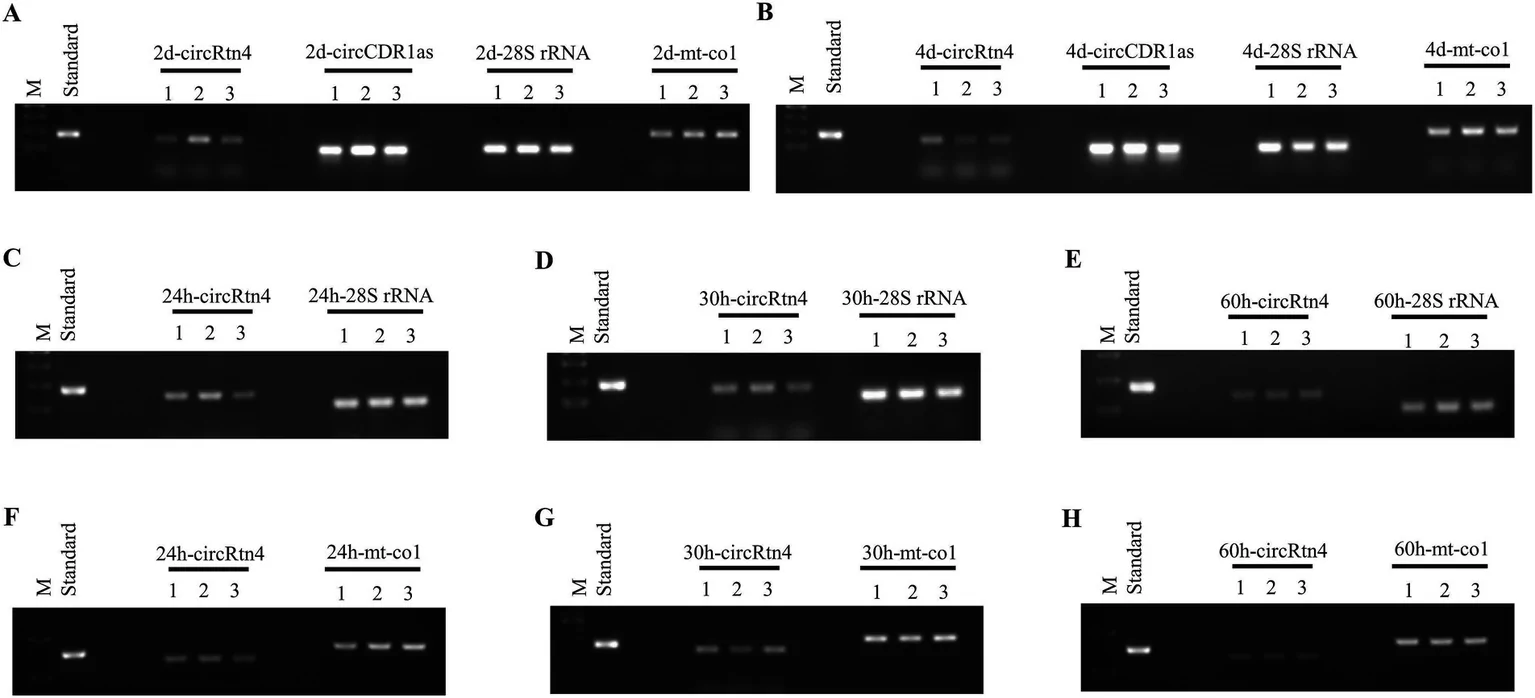
Validation of the mouse PMI estimation model by 25 °C and 35 °C equation, respectively. **(A)** The electrophoresis results of circRtn4, circCDR1as, 28S rRNA and mt-co1 of mouse samples at 2 days of 25 °C. **(B)** The electrophoresis results of circRtn4, circCDR1as, 28S rRNA, and mt-co1 of mouse samples at 4 days of 25 °C. **(C–E)** The electrophoresis results for circRtn4 matched specifically with the 28S rRNA at 35 °C for validation samples with known PMIs of 24 h, 30 h, and 60 h. **(F–H)** The electrophoresis results for circRtn4 matched specifically with the mt-co1 at 35 °C for validation samples with known PMIs of 24 h, 30 h, and 60 h.

**Table 3:** Verification of mathematical models by semi-quantitative RT-PCR.

Temperature	Internal reference	Real PMI	Estimation PMI	Estimation Error	Error rate (%)	Equation
25 °C	circCDR1as	2 d	3.5 d	1.5 d	75.0	Linear
		2 d	3.1 d	1.1 d	55.0	Quadratic
		2 d	3.1 d	1.1 d	55.0	Cubic
		4 d	4.7 d	0.7 d	17.5	Linear
		4 d	4.3 d	0.3 d	7.5	Quadratic
		4 d	4.2 d	0.2 d	5.0	Cubic
	28S rRNA	2 d	3.3 d	1.3 d	65.0	Linear
		2 d	2.7 d	0.7 d	35.0	Quadratic
		2 d	2.8 d	0.8d	40.0	Cubic
		4 d	4.3 d	0.3d	7.5	Linear
		4 d	3.6 d	0.4 d	10.0	Quadratic
		4 d	3.6 d	0.4d	10.0	Cubic
	mt-co1	2 d	2.4	0.4	20.0	Linear
		2 d	2.4	0.4	20.0	Quadratic
		2 d	1.9	0.1	5.0	Cubic
		4 d	4.4	0.4	10.0	Linear
		4 d	4.4	0.4	10.0	Quadratic
		4 d	5.9	1.9	47.5	Cubic
35 °C	28S rRNA	24 h	19.4 h	4.6 h	19.2	Linear
		24 h	19.7 h	4.3 h	18.0	Quadratic
		24 h	22 h	2 h	8.3	Cubic
		30 h	19.4 h	10.6 h	35.3	Linear
		30 h	19.7 h	10.3 h	34.3	Quadratic
		30 h	22.0 h	8.0 h	26.7	Cubic
		60 h	71.1 h	11.1 h	18.5	Linear
		60 h	71.7 h	11.7 h	19.5	Quadratic
		60 h	69.3 h	9.3 h	15.5	Cubic
	mt-co1	24 h	24.9 h	0.9 h	3.8	Linear
		24 h	23.3 h	0.7 h	2.9	Quadratic
		24 h	26.8 h	2.8 h	11.7	Cubic
		30 h	25.6 h	4.4 h	14.7	Linear
		30 h	23.9 h	6.1 h	20.3	Quadratic
		30 h	27.3 h	2.7 h	9.0	Cubic
		60 h	86.4 h	26.4 h	44.0	Linear
		60 h	87.6 h	27.6 h	46.0	Quadratic
		60 h	82.7 h	22.7 h	37.8	Cubic

d, day; h, hour.

The mathematical model of the semi-quantitative PCR experiment was validated in samples with known PMI (24 h, 30 h and 60 h) at 35 °C (Figures 5C–H). When 28S rRNA was applied as the internal reference, the error rates of the 24 h validation samples for the three equations were 19.2%, 18%, and 8.3%, respectively; the error rates of the 30 h validation samples for the three equations were 35.3%, 34.3%, and 26%, respectively; the error rates of the 60 h validation samples for the three equations were 18.5%, 19.5%, and 15.5%, respectively. When mt-co1 was used as internal reference, the error rates of the 24 h validation samples for the three equations were 3.75%, 2.9%, and 11.7%, respectively; the error rates of the 30 h validation samples for the three equations were 14.7%, 20.3%, and 9%, respectively; the error rates of the 60 h validation samples for the three equations were 44%, 46%, and 37.8%, respectively (Table 3).

The mathematical model of the RT-qPCR experiment was validated in samples with known PMI (2 d and 4 d) at 25 °C. When 28S rRNA was applied as an internal reference, the error rate of the 2 d validation samples for the three equations were 85%, 35%, 30%, respectively, and the error rate of the 4 d validation samples were 2.5%, 30%, 32.5%, respectively (Table 4).

**Table 4:** Validation of mathematical models by RT-qPCR at 25 °C.

Internal reference	Real PMI	Estimation PMI	Estimation error	Error rate (%)	Equation
28S rRNA	2 d	3.7 d	1.7 d	85.0	Linear
	2 d	2.7 d	0.7 d	35.0	Qua dratic
	2 d	2.6 d	0.6 d	30.0	Cubic
	4 d	3.9 d	0.1 d	2.5	Linear
	4 d	2.8 d	1.2 d	30.0	Qua dratic
	4 d	2.7 d	1.3 d	32.5	Cubic

d, day; h, hour.

### 3.7 Preliminary analysis of Human Samples by Semi-Quantitative RT-PCR

Following the identification of the homologous human circular RNA circRTN4 for circRtn4, homology analysis revealed a high sequence similarity of 81% between them (Figure 6A). Specific primers were designed based on the circRTN4 sequence, with details shown in Figure 6B. Expression analysis in human tissues confirmed that circRTN4 is highly expressed in brain tissue (Figure 6C). To precisely determine its back-splice junction, Sanger sequencing was performed using human brain samples. The results showed that the circRTN4 sequence matched the database data, and its splicing site was identical to that of circRtn4 (Figure 6D).

**Figure 6:**
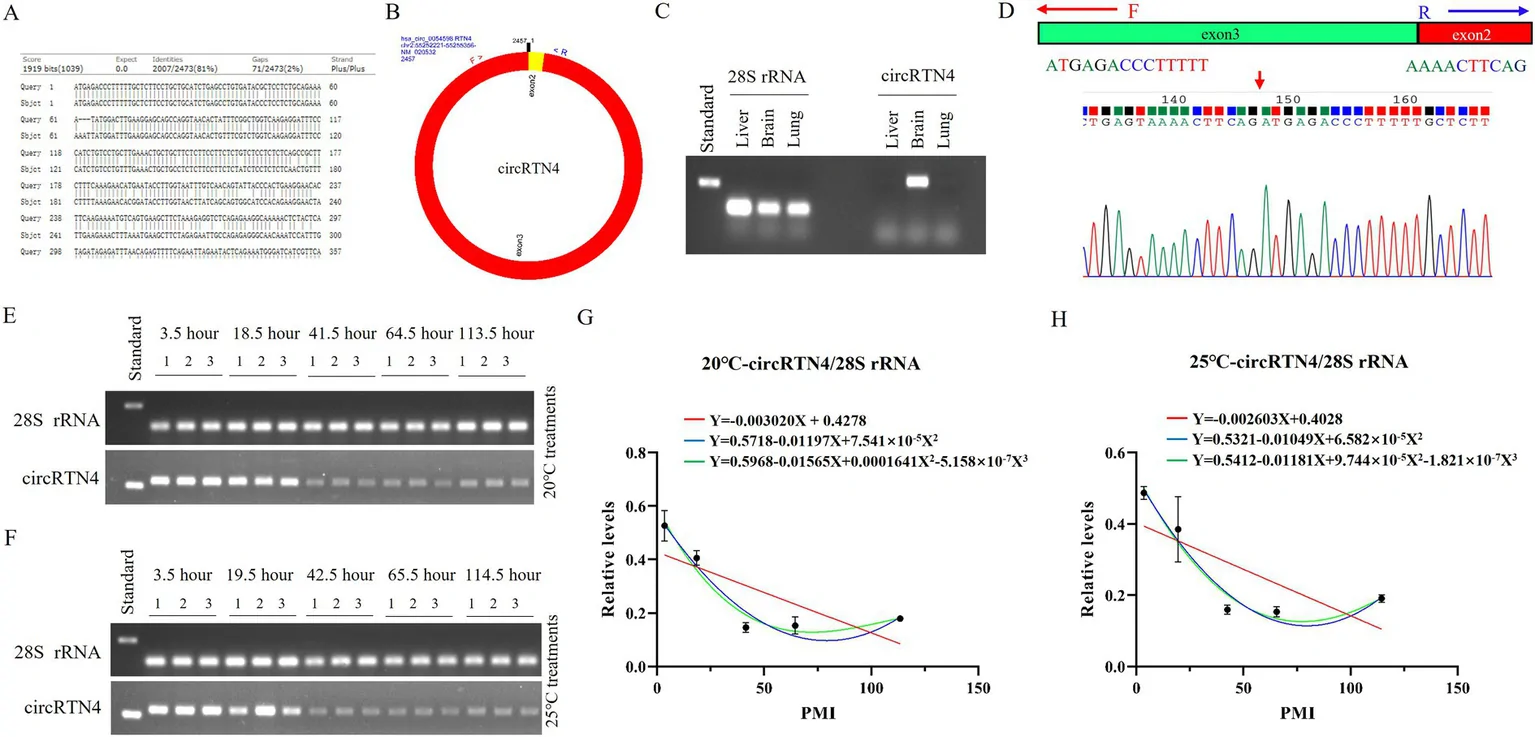
Preliminary exploratory analysis of circRTN4 in human brain tissue. **(A)** BLAST alignment results showing sequence homology between circRTN4 and circRtn4. **(B)** The CircPrimer software showed the primers positions of circRTN4. **(C)** Expression of circRTN4 and 28S rRNA in human liver, brain, and lung tissues. **(D)** Identification of circRTN4 sequences by Sanger sequencing. F, Forward primer; R, Reverse primer. Red arrows: divergent primer; Blue arrows: convergent primer. Vertical red arrow: the splice sites of exons 2 and 3 of circRTN4. **(E)** The plots of semi-quantitative RT-PCR electrophoresis results for circRTN4 and 28S rRNA at 20 °C. **(F)** The plots of semi-quantitative RT-PCR electrophoresis results for circRTN4 and 28S rRNA at 25 °C. **(G)** The linear, quadratic, and cubic equations were built at 20 °C. **(H)** The linear, quadratic, and cubic equations were built at 25 °C.

Using semi-quantitative RT-PCR, the relative expression levels of circRTN4 were measured at different PMIs under two temperature conditions, 20 °C and 25 °C (Figures 6E, F). In human brain tissue, circRTN4 showed significant degradation around 2 days postmortem, remaining at a low level thereafter. Based on the normalized relative levels data, mathematical equations (including linear, quadratic, and cubic equations) were successfully established for both 20 °C and 25 °C. The quadratic equation demonstrated higher *R*^2^ values than the corresponding linear equations, with an *R*^2^ of 0.9502 and a *p* value of 0.050 at 20 °C, and an *R*^2^ of 0.9576 and a *p*-value of 0.042 at 25 °C. More details of these exploratory regression equations are shown in Table 5.

**Table 5:** Preliminary regression equations for circRTN4 degradation using semi-quantitative RT-PCR from human samples.

Temperature	Internal reference	Regression model	Equation	*R* ^2^	*p*
20 °C	28S rRNA	Linear	Y = -0.003020X + 0.4278	0.5635	0.144
		Quadratic	Y = 0.5718–0.01197X + 0.00007541×^2^	0.9502	0.050
		Cubic	Y = 0.5968–0.01565X + 0.0001641×^2^-0.0000005158×^3^	0.9579	0.260
25 °C	28S rRNA	Linear	Y = -0.002603X + 0.4028	0.5553	0.148
		Quadratic	Y = 0.5321–0.01049X + 0.00006582×^2^	0.9576	0.042
		Cubic	Y = 0.5412–0.01181X + 0.00009744×^2^−0.0000001821×^3^	0.9589	0.256

## 4 Discussion

PMI was defined as the interval between physiological death and autopsy. The traditional method of PMI estimation based on postmortem phenomena was often inaccurate or empirical. With the development of molecular biology technology, scientists can more accurately use biomarkers such as DNA and RNA to detect PMI. However, environmental temperatures or other factors in different environments had a particularly significant effect on the degradation of molecular biomarkers. In particular, RNA is more unstable than DNA due to the abundance of ribonucleases in the environment and organisms. The postmortem environment is complex and changeable, and enzymes that can cause mRNA degradation are ubiquitous in nature, so mRNA degrades rapidly. Studies showed that mRNA may be suitable for early PMI estimation (40, 41). With the development of genetics technology, research began to focus on ncRNA, among which miRNAs were more studied. MiRNAs are tissue-specificity and less susceptible to degradation. Research showed that tissue origin, time of death, cause of death, and temperature may influence miRNA degradation (42). The ideal ncRNA markers should be characterized by specificity, sensitivity, stability, non-destructive, universality, and high efficiency (6). For these factors, it was needed to find new markers that were not susceptible to various complex factors for the establishment of PMI estimation models.

CircRNA has been a research hotspot in recent years. It can be used as a miRNA sponge to participate in the molecular diagnosis of cancer, cardiovascular diseases, and other diseases. At present, circRNA has been studied in the fields of forensic science. For example, for body fluid identification, circRNA expression profiles were used to distinguish semen, saliva, and venous blood (43). In the analysis of the cause of death, circSLC8A1 and circNFIX may serve as adjunct diagnostic markers for sudden cardiac death from acute ischemic heart disease in forensic medicine (44). CircRNAs were most highly expressed and most specific in the human brain compared with other tissues (28). CircRNAs specifically expressed in brain tissues were tested as biomarkers for this study. In the course of this study, we synthesized more than one hundred primer pairs for circRNAs, but not all circRNAs could be detected well. Only four of them were selected as potential biomarkers for our following study.

*RTN4* is related to the function of neuroendocrine secretion and neurogenesis and is homologous in humans and mice. The *Rtn4* is expressed in various tissues, including the central nervous system. Research has shown that circRtn4 is highly expressed in the mouse brain (29). As one of the circular transcripts of circRtn4, mmu_circ_0000250 was created by head-to-tail splicing of exon 2 and exon 3 of the *Rtn4*, with a total length of 2,418 base pairs. Similarly, hsa_circ_0054598 was created by head-to-tail splicing of exon 2 and exon 3 of the *RTN4*, with a total length of 2,457 base pairs. Currently, circRtn4 has been extensively studied in physiology and disease. For example, in terms of physiology, mmu_circ_0000250/circRtn4 promotes neuronal development and can act as a miR-24-3p sponge that promotes neurite growth by regulating target gene CHD5 (chromodomain helicase DNA binding protein 5) (45). In terms of disease, Rtn4-Exos sponges miR-146a to attenuate TNF-α-induced cytotoxicity and apoptosis and treats TNF-α-induced osteoporosis, thus Rtn4-Exos may be a drug candidate (46). Through absorption of miR-128-3p/SIRT1 in diabetes, mmu_circ_0000250/circRtn4 can strengthen the therapeutic effect of adipose-derived mesenchymal stem cells-exosomes to boost wound healing (47). Therefore, mmu_circ_0000250/circRtn4 was expected to be a potential biomarker for PMI estimation and used to establish mouse PMI estimation models. Based on its human homologous circRNA (Hsa_circ_0054598/circRTN4), exploratory regression equations were fitted. It is further hoped that these models can be used for investigation of human samples.

Moreover, it is important to explore the internal references that are stable after death for the construction of the PMI estimation model. During the screening internal references, we selected ten reference biomarkers as the potentials, including seven mRNAs, two rRNAs, one mitochondrial mRNA, and one circRNA. On the ICG website, these seven mRNAs showed high usage frequency in human studies. In the relevant literature, 28SrRNA, 18S rRNA, and 5S rRNA were often used as candidate reference genes in PMI estimation (8, 9). Literatures showed that 18S rRNA degradation is slow and can still be detected at about 7 d, and 28S rRNA in dental pulp could still be detected after 45 d (38). As for 5S rRNA, one study found that the amplification efficiency is too low and may reduce the sensitivity of the reference gene, so 5S rRNA is not used as a reference gene in our study (26). Because 28S rRNA, mt-co1, and circCDR1as represent different RNA classes, differences in extraction recovery, reverse-transcription efficiency, and postmortem degradation kinetics may introduce reference-dependent normalization effects. In the present study, these RNAs were evaluated and used separately rather than combined into a common normalization factor. Although the same RNA extraction and reverse-transcription procedures were applied to all samples, this design cannot fully eliminate RNA-class-specific analytical bias.

The expression patterns of 13 circRNAs and their linear isoforms were examined in six anatomical brain regions of the mouse, circCDR1as can be used as a positive control in their study (29). So, we also selected the commonly used reference genes such as 18S rRNA, 28S rRNA, and circCDR1as in the study. Additionally, this study explored the potential of mt-co1 as a reference gene for these models. In the estimation of PMI, most housekeeping genes were found to degrade rapidly with PMI. The details of semi-quantitative RT-PCR showed that mRNA was degraded rapidly with PMI and most of them degraded rapidly after 2 d. Therefore, mRNAs are less stable after death and are usually not suitable as an internal reference. According to the experimental results, 28S rRNA, 18S rRNA, mt-co1 and circCDR1as were found to degrade slowly over time and were more stable than mRNA. However, the results of semi-quantitative RT-PCR showed that 18S rRNA was rapidly degraded after 4 d and was not suitable as a reference gene at 25 °C and circCDR1as was rapidly degraded at 35 °C. Because mt-co1 is more stable than circCDR1as and similar to the stability of 28S rRNA, we chose mt-co1 as the reference gene at 35 °C. Finally, we screened out 28S rRNA, mt-co1 and circCDR1as as the internal references at 25 °C, and 28S rRNA and mt-co1 as the internal references at 35 °C in this study to establish the equation.

We obtained the corresponding *p*-values, *R*^2^ values, and error rates for the equations established at different temperatures. The closer the *R*^2^-value is to 1, the better to fit the model. The significance of the *p* value is to determine whether the fitted curve is sufficiently reliable and whether the model reflects the characteristics of the data well. However, the *p*-value may be affected by the number of samples and the complexity of the fitted model, so it is necessary to combine the validation results of the model for comprehensive analysis. In the semi-quantitative RT-PCR experiment at 25 °C, the K values of circCDR1as, 28S rRNA and mt-co1 were 0.5716, 0.6718, and 0.9055, respectively. The *R*^2^-value was the lowest in the linear equation, and the *R*^2^ values were higher in the quadratic and the cubic equations, and the *p*-values of the linear equation and the quadratic equation were less than 0.05 at 25 °C. At 35 °C conditions, the *K*-value of 28S rRNA and mt-co1 was 0.4475 and 0.5455 respectively, and the *R*^2^-values of all three equations were close to 0.96–0.99 and the *p*-values were less than 0.05. Although higher-order equations produced higher R^2^-values, the additional model-comparison analyses showed that increasing polynomial complexity did not consistently improve predictive performance. Quadratic models were supported for the 25 °C data normalized to 28S rRNA and circCDR1as, whereas linear models were favored for mt-co1 at 25 °C and for both reference RNAs at 35 °C. Cubic terms provided no additional benefit. These findings emphasize that model selection should consider model complexity and cross-validated predictive performance rather than R^2^ alone. In the real-time RT-PCR experiment at 25 °C, the R^2^ value of the linear equation calculated from the Ct values was also the lowest. The *R*^2^ values were higher for the quadratic equation and cubic equation, but only the *p*-value of the linear equation was less than 0.05. These results suggested that the non-linear equation model needed to be further optimized. According to the error rate of the linear equation, the longer the PMI, the more accurate the estimation was likely to be. For the quadratic equation and the cubic equation, we note that they have a similar error rate of about 30% in the PMI estimation for 2 d and 4 d. The validation of the present models remains preliminary. Only a limited number of known-PMI samples and validation time points were available. The validation was therefore insufficient to establish generalizable predictive accuracy or practical forensic applicability. Future studies should use larger independent cohorts, broader PMI and temperature ranges, prospectively defined training and validation sets, blinded prediction procedures, and prediction-oriented statistics with appropriate uncertainty estimates. In fact, we also explored the degradation of circRtn4 at 4 °C, but the *p* and R^2^ values of the constructed equations were not ideal. Therefore, under low-temperature conditions, it may not be an optimal circRNA biomarker. However, in another study of ours, a PMI estimation model was constructed at low temperatures based on circRNAs from liver tissue (48). The study suggests that circRNAs from different tissues may play distinct roles in PMI estimation.

We should point out that the samples of 16-week-old mice were collected at 25 °C, while the samples of 9-week-old mice were collected at 35 °C in this study. Studies have shown that circRNA is developmental-stage specific and closely related to neurodevelopment (49, 50). Because circRNA expression and metabolism may vary with developmental stage, differences between the two model sets cannot be attributed solely to temperature. The current analyses should therefore be interpreted as condition-specific rather than as an independent assessment of temperature effects. The difference in the age of the mice may make the K values of the two estimation models of temperature different. Therefore, it may be better if we divide the sample into different age groups to build the estimation model. Our results also suggested that more relevant factors, such as age and sex, needed to be taken into account when constructing brain tissue models. In addition, compared to more advanced quantitative platforms such as RT-qPCR or digital PCR (dPCR), semi-quantitative RT-PCR is characterized by a narrower dynamic range and is more vulnerable to band saturation, imaging variability, and operator-dependent quantification. Although we mitigated these issues by incorporating a standard DNA sample on every gel to normalize exposure conditions and strictly controlling the analytical parameters via ImageJ, RT-qPCR and dPCR remain the gold standards for exact molecular quantification. Nevertheless, semi-quantitative RT-PCR, which separates PCR products by gel electrophoresis, is straightforward to perform, requires only basic instrumentation, and incurs relatively low experimental costs. Furthermore, gel electrophoresis provides an intuitive visual assessment of postmortem RNA degradation. The fact that PMI estimation models could be established using both semi-quantitative RT-PCR and RT-qPCR suggests that the postmortem degradation pattern of circRtn4 is robust enough to be captured by standard laboratory techniques. However, future casework translations should preferably rely on RT-qPCR or dPCR to ensure maximum predictive accuracy.

Although the controlled mouse model enabled the systematic evaluation of postmortem circRNA changes, its direct extrapolation to other animals or humans remains limited. Because the human data were derived from a single healthy male donor in this study, it cannot account for the biological variations encountered in forensic science, such as age, sex, diverse causes of death, and environmental conditions. Therefore, the equations generated from the human sample do not constitute a validated predictive model. The human analysis revealed that circRTN4 holds potential for PMI estimation. However, postmortem RNA dynamics may differ among species and may also be influenced by environmental conditions. In future studies, the PMI estimation model will be refined by collecting additional samples for the application of veterinary forensic science.

In addition, it contributed to improving the accuracy of the estimation model by screening more circRNA markers and more stable internal reference genes. In future studies, the combined application of multi-tissue circRNAs as well as the exploration of circRNA changes at more temperatures and time points after death may lead to the construction of more appropriate PMI estimation models. From a veterinary thanatology perspective, validation in additional animal species will also be essential to determine how species-specific physiology and environmental exposure influence postmortem circRNA degradation. Such studies may facilitate the development of species- and temperature-specific molecular approaches to PMI estimation.

## 5 Conclusion

CircRtn4 might be an effective molecular marker for PMI estimation in mouse brain tissue. The degradation of mRNA is time-dependent and not suitable for internal control. 28S rRNA and circCDR1as are suitable as reference genes for the mathematical model, and mt-co1 is suitable as a reference gene at high temperature. Model comparison indicated that the preferred regression form depended on the reference RNA and experimental condition, and increasing polynomial complexity did not consistently improve predictive performance. Furthermore, our exploratory analysis of the homologous circRTN4 in a single human sample provides an initial proof-of-concept for its translational potential. However, it must be emphasized that these findings are strictly exploratory. Taken together, we explored the application of circRtn4/circRTN4 in the possible PMI estimation, offering preliminary evidence for cross-species candidate markers. Further validation in independent cohorts and additional animal species is required before their application in human or veterinary forensic casework.

## Data Availability

The raw data supporting the conclusions of this article will be made available by the authors, without undue reservation.

## Footnotes

1
http://circbase.org

2
http://www.circbank.cn/

3
http://gb.whu.edu.cn/TSCD/

4
https://www.ncbi.nlm.nih.gov/

5
https://commonfund.nih.gov/GTEx

6
https://ngdc.cncb.ac.cn/icg/

7
https://blast.ncbi.nlm.nih.gov/Blast.cgi
